# First-line treatment with KN046, chemotherapy and palliative radiotherapy for advanced esophageal squamous cell carcinoma: an open-label, dose escalation, and dose expansion phase Ib trial

**DOI:** 10.1007/s00262-024-03769-4

**Published:** 2024-08-06

**Authors:** Qi Zhao, Xi Su, Jiao Xue, Yandong Liu, Jiaxing Zhu, Xuwei Cai, Songbing Qin

**Affiliations:** 1 Department of Radiation Oncology, The First Affiliated Hospital of Soochow University, Suzhou, 215000 China; 2 Department of Radiation Oncology, Shanghai Chest Hospital, School of Medicine, Shanghai Jiao Tong University, Shanghai, 200030 China

**Keywords:** Esophageal squamous cell carcinoma, Chemoradiotherapy, Immune checkpoint inhibitors, Bispecific antibodies

## Abstract

There is growing evidence to suggest that radiotherapy might enhance the efficacy of immunotherapy. This study aimed to assess the possibility of KN046, a bispecific antibody targeting PD-L1 and CTLA-4, combined with chemotherapy and palliative radiotherapy for advanced esophageal squamous cell carcinoma (ESCC). In this open-label, phase Ib trial, patients with advanced ESCC were administered chemotherapy with palliative radiotherapy, and KN046 in the predefined escalation dosages of 1, 3, or 5 mg/kg (every 3 weeks during chemotherapy cycles and every 2 weeks during KN046 maintenance). The chemotherapy regimen constituted cisplatin (75 mg/m^2^ i.v., d1) and paclitaxel (135–175 mg/m^2^ ivgtt., d1). Radiotherapy specifics, including site, timing, dose, and fragmentation pattern, were at the investigator’s discretion. The primary outcome was dose-limiting toxicity (DLT). From May 2019 to April 2021, 25 patients were enrolled across the dosage groups: 3 in 1 mg/kg, 12 in 3 mg/kg, and 10 in 5 mg/kg. No DLT was observed during the dose escalation. The objective response rate was 41.7% (95%CI 22.1–63.4), while the disease control rate was 87.5% (95%CI 67.6–97.3). At a median follow-up of 11.8 months, the median progression-free survival was 7.8 months (95%CI 5.2–9.7) and median overall survival was 15.9 months (95%CI 8.4-NE). Serious adverse events were reported in 48.0% of patients, predominantly leukopenia (16%), immune-mediated enterocolitis (12%), immune-mediated pneumonitis (8%), and neutropenia (8%). Combining KN046 with chemotherapy and palliative radiotherapy might be feasible, showing a favorable safety profile and notable efficacy in advanced ESCC patients.

**Supplementary Information:**

The online version contains supplementary material available at 10.1007/s00262-024-03769-4.

## Introduction

Efficacious treatment modalities for esophageal squamous cell carcinoma (ESCC) are demanded, since the standard first-line recommendation of 5-fluorouracil and platinum-based chemotherapy have offered limited benefit, often restricting overall survival (OS) to under a year [1]. Robust evidence from the KEYNOTE-590 [2] and CheckMate 648 trials [3] underlined the potency of concomitant administration of immunotherapy with chemotherapy. The CheckMate 648 trial further demonstrated the potential of dual immunotherapy targeting both programmed death ligand-1 (PD-L1) and cytotoxic T lymphocyte-associated protein 4 (CTLA-4) in advanced ESCC [3], as well as in other neoplasms [4, 5].

Radiotherapy has garnered interest in the realm of oncology for its potential synergistic effects with immunotherapy [6, 7]. Central to this hypothesis is the abscopal effect, a principle wherein local radiotherapy elicits systemic anti-tumor responses beyond the irradiated region [8, 9]. Mechanistically, radiotherapy instigates a cascade of cellular events. It augments the systemic liberation of tumor antigens, which are subsequently intercepted by antigen-presenting cells. These cells, in turn, prime and activate T lymphocytes, notably the CD8 cytotoxic subset, inducing a holistic immune offensive against tumor tissues [10]. Notably, radiotherapy modulates the tumor microenvironment, making it more receptive to T cell infiltration, and curtails immunosuppressive signaling pathways [11]. Clinical studies have underscored enhanced therapeutic outcomes when integrating radiotherapy with immune checkpoint inhibitors in non-small cell lung cancer, pancreatic cancer, and head and neck squamous cell carcinoma [12–14].

KN046 emerges as a pioneering humanized bispecific antibody that dually targets PD-L1 and CTLA-4, which has demonstrated a remarkable capacity to potentiate the immune response specifically within the tumor [15–17]. The first-in-human trial of KN046 (KN046-AUS-001) underlined its promising safety metrics, with a solitary case of dose-limiting toxicity (DLT) recorded at a 5.0 mg/kg dose level, administered bi-weekly [18]. Though the combination of immunotherapy and chemotherapy is currently recommended as first-line therapy for advanced ESCC, it was not standard therapeutic option when this study initiated and the benefit of dual immunotherapy is still under investigation. Furthermore, the utilization of immunotherapy to maintain the disease control from chemotherapy and the addition palliative radiotherapy before immunotherapy to enhance the anti-tumor effect was worth exploring. Hence, this phase I trial, characterized by dose-escalation and expansion phases, sought to investigate the possibility of treatment modality of combining standard chemotherapy, palliative radiotherapy, and subsequent KN046 administration in the first-line treatment against advanced ESCC.

## Methods

### Study design and participants

In this open-label, phase Ib dose-escalation and expansion study, we enrolled patients with advanced ESCC. To qualify for inclusion, participants needed to meet the following primary criteria: they had to be at least 18 years of age; possess a histological confirmation of advanced ESCC with indications for radiotherapy; have not undergone prior systemic treatment for their advanced ESCC condition; and have an Eastern Cooperative Oncology Group (ECOG) performance status (PS) of 0–1. Key exclusion criteria encompassed the presence of untreated active cerebral or meningeal metastases; any prior radical radiotherapy carried out within three months before enrollment or palliative radiotherapy within the preceding 2 weeks. A comprehensive list of inclusion and exclusion criteria is documented in Supplementary Table S1.

The study received approval from our Hospital’s Ethics Committee on May 16, 2019, and secured its registration at the Clinical Registry. The study was conducted in accordance with the Declaration of Helsinki, and all participants provided written informed consent before the study’s commencement.

### Procedure

**Fig. 1:**
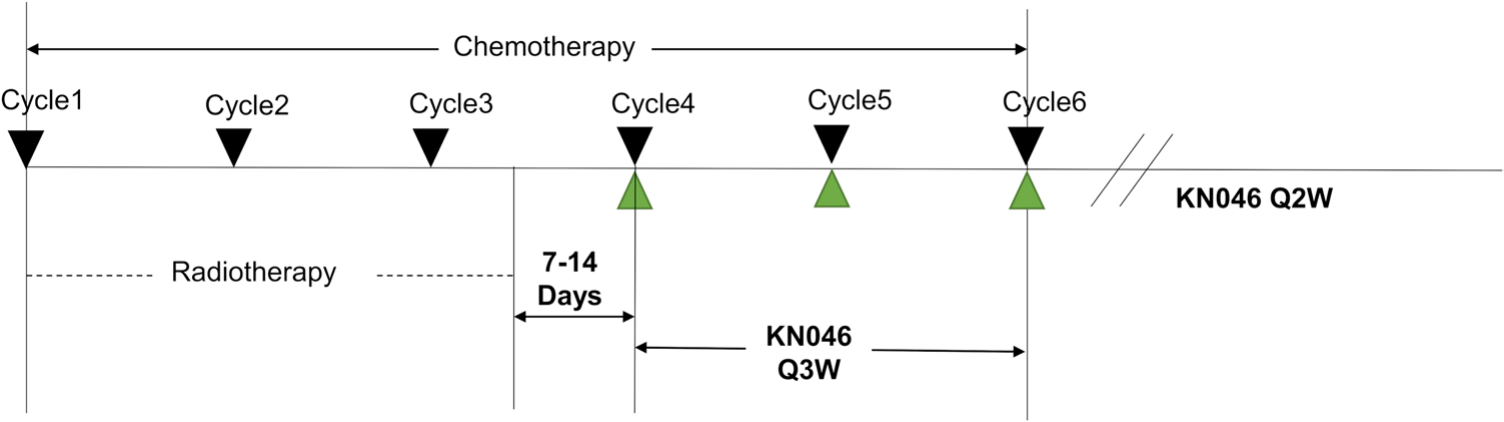
Treatment procedures. Q3W: every 3 weeks. Q2W: every 2 weeks

In our study, all participants received chemotherapy plus palliative radiotherapy and KN046, as illustrated in Fig. 1. Initially, participants underwent 4–6 chemotherapy cycles (spanning 21 days per cycle) using a regimen of cisplatin (75 mg/m^2^ i.v., day 1) and paclitaxel (135–175 mg/m^2^ ivgtt. day 1). The specific number of chemotherapy cycles was tailored to each participant, principally determined by their individual tolerability. During the first to third cycles of chemotherapy, individualized palliative radiotherapy was employed concurrently. In our phase Ib trial, radiation therapy was tailored according to the clinical presentation and history of prior treatment in patients with advanced ESCC: (1) For patients with mediastinal regional recurrence and a previous history of radiotherapy, palliative radiotherapy was administered using conventional fractionation, with total doses ranging from 30 to 40 Gy delivered in 15 to 20 fractions. (2) For patients diagnosed initially with advanced disease who had no history of surgery or radiotherapy, and who presented with regional lymph node involvement or distant metastases, a more intensive regimen was employed. These patients received a total of 50 Gy, delivered in 25 fractions, targeting the primary tumor foci and regional lymph nodes. (3) For patients with non-mediastinal regional recurrence or metastasis, the choice of radiotherapy was guided by patient tolerability. Suitable candidates underwent stereotactic body radiation therapy. For those who could not tolerate SBRT, conventional fractionated radiotherapy was provided, with doses ranging from 30 to 40 Gy delivered in 15 to 20 fractions.

Subsequent to the palliative radiotherapy, within a 7–14 day window, participants were introduced to the KN046 therapy. The administration was intravenous and dosages were set at predetermined levels of 1, 3, or 5 mg/kg, escalated based on the modified toxicity probability interval (mTPI)-2 method [19]. Throughout the chemotherapy sessions, KN046 was administered at three-week intervals. Once chemotherapy concluded, the administration frequency of KN046 shifted to every two weeks, continuing until any of the following occurred: disease progression, intolerable side effects, voluntary study exit, death, or a treatment duration reaching 108 weeks.

### Endpoints

The primary endpoint of this study was DLT, which was assessed within 21 days of the first administration of KN046. DLT evaluation encompassed both hematologic and non-hematologic toxicities, with detailed criteria available in Supplementary Table S1.

Efficacy endpoints included the overall response rate (ORR), disease control rate (DCR), progression-free survival (PFS), and OS. ORR was defined as the proportion of participants achieving complete response (CR) or partial response (PR). DCR was defined as the proportion achieving CR, PR, or stable disease (SD). PFS was the time calculated from enrollment to disease progression or death from any cause, while OS was the time from enrollment to death from any cause. Disease progression was assessed based on Response Evaluation Criteria in Solid Tumors (RECIST) 1.1 criteria. Efficacy evaluations occurred every 6 weeks during the first year of treatment and every 12 weeks thereafter.

Safety endpoints encompassed treatment-emergent adverse events (TEAEs), KN046-related TEAEs, and serious adverse events (SAEs) following the Common Terminology Criteria for Adverse Events of the National Cancer Institute (NCI-CTCAE) Version 5.0. Follow-up visits for TEAEs extended until 30 days after the last KN046 administration or initiation of a new antitumor treatment, whichever came first. SAEs and KN046-related TEAEs were tracked up to 90 days after the last administration.

Stool samples were collected prior to KN046 administration. Metabolomics analysis was performed using liquid chromatography-mass spectrometry (LC–MS) on the Thermo Ultimate 3000 platform equipped with ACQUITY UPLC^®^ HSS T3 1.8 µm columns. The samples were subjected to analysis using an electrospray ionization (ESI) mass analyzer, specifically the Thermo Q Exactive Plus model.

### Statistical analysis

Due to the exploratory nature of this study, no hypothesis was tested. The sample size for the dose-escalation phase was determined using the mTPI-2 method. Approximately 20 to 30 patients were planned to be enrolled in the dose expansion phase to capture any additional safety or efficacy signals, although no predefined statistical hypotheses were established. The safety set (SS) consisted of all subjects who received KN046 at least once. The efficacy analysis set (EAS) included all subjects who received KN046 at least once and underwent at least one efficacy evaluation.

PFS and OS rates were calculated using the Kaplan–Meier method. The ORR and DCR were calculated based on the EAS, and their corresponding 95% CIs were calculated using the Clopper-Pearson method. For biomarker analysis, data processing was performed using ProteoWizard (v3.0.8789) and R, followed by matching with the Biodeep’s mass spectrometry database. Short-chain fatty acid (SCFA) data were presented as means ± standard errors. Multivariate data analysis included principal component analysis (PCA) and orthogonal partial least squares discriminant analysis (OPLS-DA). To compare metabolites between participants with different treatment responses, the Mann–Whitney-Wilcoxon test was utilized. MetaboAnalyst was employed to conduct metabolic pathway topology analysis of the metabolites. Receiver operating curve (ROC) analysis was performed to assess the potential of treatment response prediction, with the area under the curve (AUC) being calculated. Significant enrichment was indicated by raw *P* values (Raw*P*) < 0.05 and impact values > 0.

## Results

### Baseline characteristics of the participants

A total of 25 participants were enrolled in the study, with three cases receiving a dose of 1 mg/kg, 12 participants receiving 3 mg/kg, and 10 participants treated with 5 mg/kg. The median age of the participants was 67.0 years (range: 52–75 years). Among the enrolled participants, 7 (28.0%) had newly diagnosed metastasis disease and 18 (72.0%) had recurrent disease. Besides, 16 patients (64.0%) had a history of prior radiotherapy treatment and 11 patients (44.0%) had undergone prior surgery. Comprehensive baseline characteristics of the participants are provided in Table 1. The data cutoff for the study was April 30, 2021.

**Table 1:** Baseline characteristics of the participants

	All (n = 25)	1 mg/kg (n = 3)	3 mg/kg (n = 12)	5 mg/kg (n = 10)
Age, years, median (range)	67.0 (52, 75)	67.0 (66, 70)	67.5 (52, 75)	66.0 (61, 73)
Male, n (%)	19 (76.0)	3 (100.0)	8 (66.7)	8 (80.0)
ECOG score of 1, n (%)	20 (80.0)	2 (66.7)	8 (66.7)	10 (100.0)
*Disease status at trial entry, n (%)*				
Metastatic	7 (28.0)	1 (33.3)	2 (16.7)	4 (40.0)
Recurrence, locoregional or distant	18 (72.0)	2 (66.7)	10 (83.3)	6 (60.0)
*Recurrence disease (locoregional or distant) at trial entry, n (%)*	18	2	10	6
Mediastinal region only	8 (44.4)	2 (100.0)	4 (40.0)	2 (33.3)
Mediastinal region and distant metastases	3 (16.7)	0	3 (30.0)	0
Distant metastases only	7 (38.9)	0	3 (30.0)	4 (66.7)
*Metastatic disease at trial entry, n (%)*	7	1	2	4
Lung	1 (14.3)	0	0	0
Nonregional lymph	5 (71.4)	1 (100.0)	2 (100.0)	2 (50.0)
Liver	1 (14.3)	0	0	1 (25.0)
Prior surgery, n (%)	11 (44)	1 (33.3)	5 (41.7)	5 (50.0)
Prior radiotherapy, n (%)	16 (64.0)	1 (33.3)	11 (91.7)	4 (40.0)
Prior chemotherapy, n (%)	16 (64.0)	1 (33.3)	10 (83.3)	5 (50.0)

ECOG: Eastern cooperative oncology group

### Safety

The median duration of KN046 administration varied across the dosage groups: 33.1 weeks (range: 13.7–38.0) for the 1 mg/kg group, 19.8 weeks (range: 3.0–84.0) for the 3 mg/kg group, and 23.3 weeks (range: 3.0–51.0) for the 5 mg/kg group.

No DLTs were observed during the dose escalation phase, and the AEs that occurred during this period are detailed in Supplementary Table S3. Among the 25 enrolled patients, 24 individuals (96%) reported TEAEs. The most prevalent TEAEs included leukopenia (n = 20, 80.0%), neutropenia (n = 16, 64.0%), and nausea (n = 10, 40.0%) as shown in Table 2. The incidence rate of grade ≥ 3 TEAEs was 60% (n = 15), with the most common being leukopenia (n = 13, 52.0%) and neutropenia (n = 12, 48.0%, Table 2).

**Table 2:** Adverse events

	All (n = 25)		1 mg/kg (n = 3)		3 mg/kg (n = 12)		5 mg/kg (n = 10)	
	Any grade	Grade ≥ 3	Any grade	Grade ≥ 3	Any grade	Grade ≥ 3	Any grade	Grade ≥ 3
Any TEAEs	24 (96.0)	15 (60.0)	3 (100.0)	3 (100.0)	12 (100.0)	6 (50.0)	9 (90.0)	6 (60.0)
KN046-related TEAE	13 (52.0)	4 (16.0)	1 (33.3)	1 (33.3)	6 (50.0)	2 (16.7)	6 (60.0)	1 (10.0)
SAE	12 (48.0)	3 (100.0)	6 (50.0)	3 (30.0)				
KN046-related SAE	6 (24.0)	1 (33.3)	2 (16.7)	3 (30.0)				
KN046-related treatment discontinuation	4 (16.0)	1 (33.3)	2 (16.7)	1 (10.0)				
*Most common TEAEs*								
Leukopenia	20 (80.0)	13 (52.0)	3 (100.0)	2 (66.7)	9 (75.0)	5 (41.7)	8 (80.0)	6 (60.0)
Neutropenia	16 (64.0)	12 (48.0)	3 (100.0)	3 (100.0)	7 (58.3)	5 (41.7)	6 (60.0)	4 (40.0)
Nausea	10 (40.0)	0	3 (100.0)	0	5 (41.7)	0	2 (20.0)	0
Thrombocytopenia	9 (36.0)	0	2 (66.7)	0	3 (25.0)	0	4 (40.0)	0
Cough	8 (32.0)	0	1 (33.3)	0	5 (41.7)	0	2 (20.0)	0
Decreased appetite	8 (32.0)	0	1 (33.3)	0	5 (41.7)	0	2 (20.0)	0
*Most common KN046-related TEAE*								
Pruritus	4 (16.0)	0	0	0	2 (16.7)	0	2 (20.0)	0
Immune-mediated enterocolitis	3 (12.0)	2 (8.0)	1 (33.3)	1 (33.3)	1 (8.3)	1 (8.3)	1 (10.0)	0
Rash	3 (12.0)	0	0	0	1 (8.3)	0	2 (20.0)	0
Dermatitis allergic	2 (8.0)	0	0	0	1 (8.3)	0	1 (10.0)	0
Immune-mediated pneumonitis	2 (8.0)	1 (4.0)	0	0	2 (16.7)	1 (8.3)	0	0
Infusion related reaction	2 (8.0)	0	0	0	1 (8.3)	0	1 (10.0)	0
*Most common SAEs*								
Leukopenia	4 (16.0)	0	3 (25.0)	1 (10.0)				
Immune-mediated enterocolitis	3 (12.0)	1 (33.3)	1 (8.3)	1 (10.0)				
Immune-mediated pneumonitis	2 (8.0)	2 (66.7)	0	0				
Neutropenia	2 (8.0)	1 (33.3)	1 (8.3)	0				
*Most common KN046-related SAE*								
Immune-mediated enterocolitis	3 (12.0)	1 (33.3)	1 (8.3)	1 (10.0)				
Immune-mediated pneumonitis	2 (8.0)	0	2 (16.7)	0				
Immune-mediated hepatitis	1 (4.0)	0	0	1 (10.0)				
Rash	1 (4.0)	0	0	1 (10.0)				

All data were presented as n (%)

TEAE, treatment-emergent adverse events; SAE, serious adverse event

SAEs were encountered by 12 participants (48%). The leading SAEs included leukopenia (n = 4, 16%), immune-mediated enterocolitis (n = 3, 12%), immune-mediated pneumonitis (n = 2, 8%), and neutropenia (n = 2, 8%). The incidence rate of KN046-related SAEs was 24.0% (n = 6), with immune-mediated enterocolitis (n = 3, 12%) and immune-mediated pneumonitis (n = 2, 8%) being the most prevalent. A total of four participants (16.0%) had to discontinue treatment due to KN046-related TEAEs, which included two cases of immune-mediated enterocolitis, one case of immune-mediated pneumonitis, and one case of immune-mediated hepatitis.

### Efficacy

**Fig. 2:**
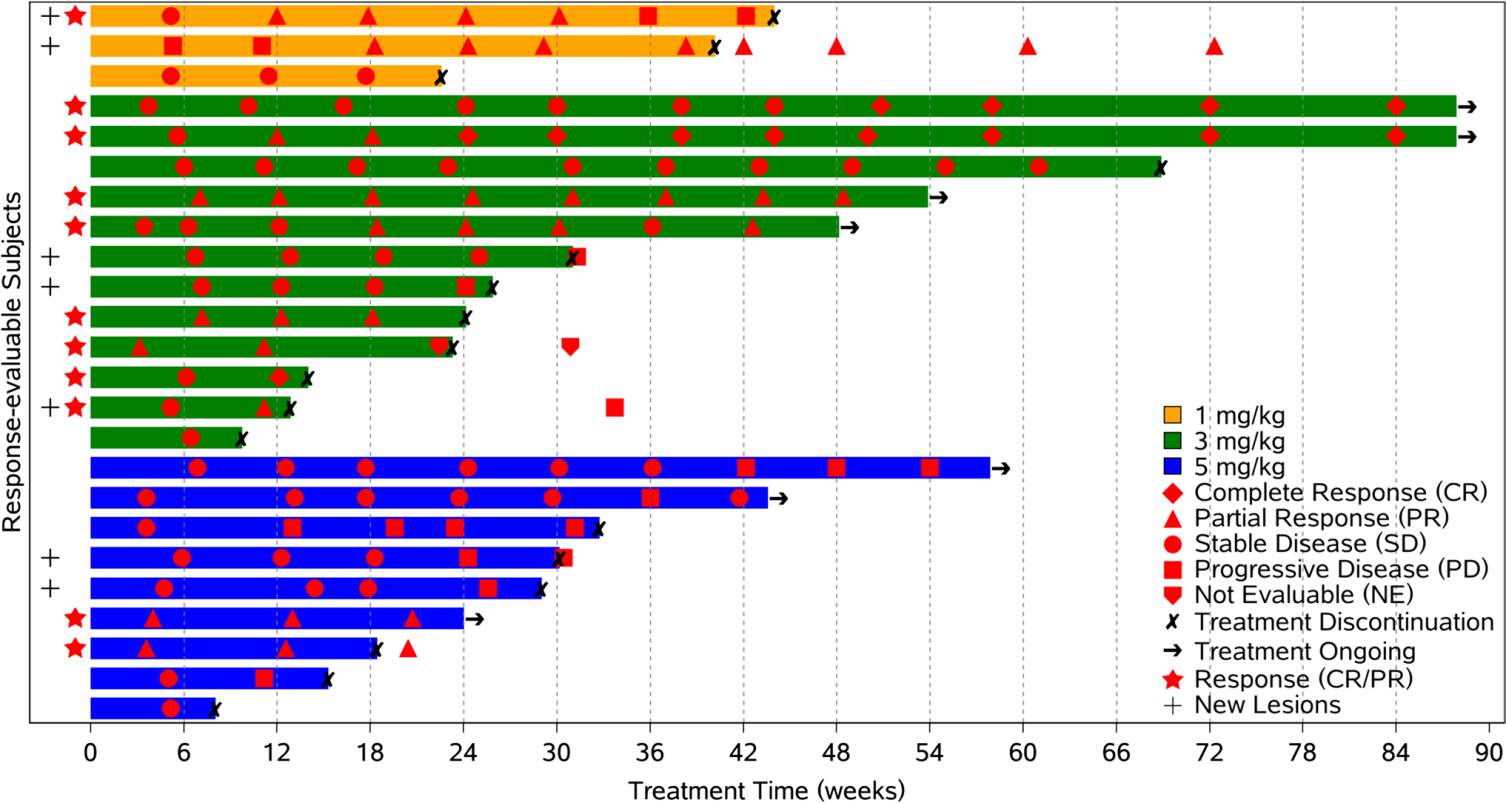
Swimming lane plot of treatment response. CR: complete response; PR: partial response; SD: stable disease; PD: progressive disease; NE: non-evaluable

One participant in the 5 mg/kg group could not be evaluated for efficacy since they did not receive KN046 after completing only one cycle of chemotherapy due to intolerability. The treatment response is illustrated in Fig. 2. Three participants achieved CR, and seven achieved confirmed PR, resulting in an ORR of 41.7% (95% CI 22.1%-63.4%). The DCR was 87.5% (95% CI 67.6%-97.3%), as shown in Table 3.

**Table 3:** Efficacy

	All (n = 24)	1 mg/kg (n = 3)	3 mg/kg (n = 12)	5 mg/kg (n = 9)
ORR	41.7 (22.1–63.4)	33.3 (0.8–90.6)	58.3 (27.7–84.8)	22.2 (2.8–60.0)
DCR	87.5 (67.6–97.3)	66.7 (9.4–99.2)	100.0 (73.5–100.0)	77.8 (40.0–97.2)
CBR	58.3 (36.6–77.9)	33.3 (0.8–90.6)	75.0 (42.8, 94.5)	44.4 (13.7–78.8)
Median PFS	7.8 (5.2, 9.7)	5.2 (1.2, NE)	NE (5.6, NE)	5.6 (2.6, 8.3)
6 month PFS rate	57.0 (33.2, 75.1)	33.3 (0.9, 77.4)	80.8 (42.3–94.9)	31.1 (4.6–64.1)
9 month PFS rate	34.2 (14.5, 55.1)	0	57.7 (22.1, 81.9)	15.6 (0.8, 49.1)
12 month PFS rate	28.5 (10.7, 49.5)	0	57.7 (22.1, 81.9)	0 (NE, NE)
Median OS	15.9 (8.4, NE)	11.1 (5.2, NE)	NE (7.2, NE)	NE (5.0, NE)
6 month OS rate	87.1 (64.9, 95.6)	66.7 (5.4, 94.5)	91.7 (53.9, 98.8)	87.5 (38.7, 98.1)
9 month OS rate	73.3 (49.9, 87.1)	66.7 (5.4, 94.5)	75.0 (40.8, 91.2)	72.9 (27.6, 92.5)
12 month OS rate	66.6 (41.6, 82.9)	33.3 (0.9, 77.4)	75.0 (40.8, 91.2)	72.9 (27.6, 92.5)

All data were presented as % (95% CI), except for median PFS and OS (months, 95%CI)

ORR, objective response rate; DCR, disease control rate; CBR, clinical benefit rate; PFS, progression-free survival; OS, overall survival; NE, non-evaluable

**Fig. 3:**
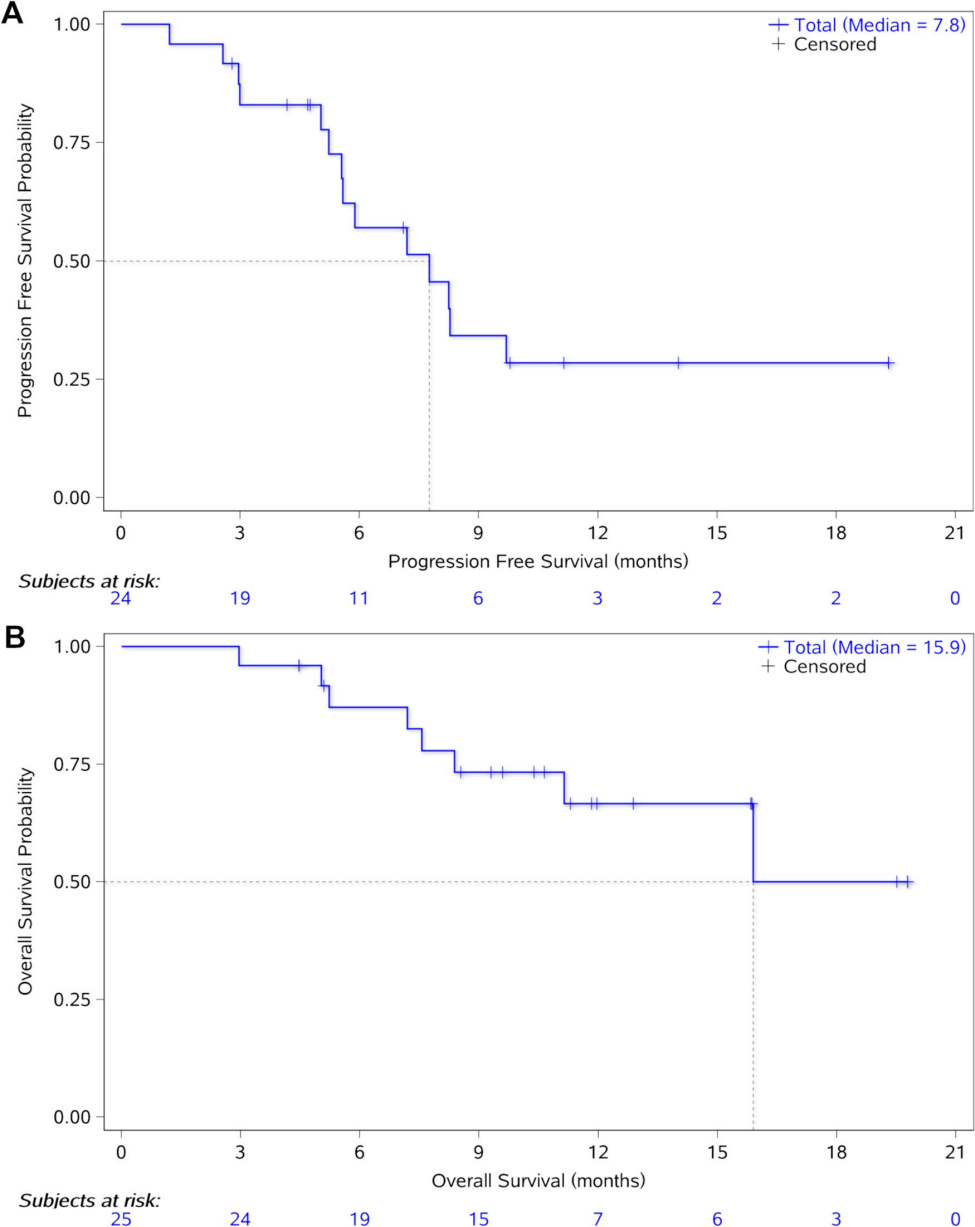
Progression-free survival **A** and overall survival **B**. NE: non-evaluable

The median follow-up was 11.8 months (range: 9.3–15.6). In the entire participant cohort, the median PFS was 7.8 months (95% CI 5.2–9.7) (Fig. 3A). This included specific median PFS durations of 5.2 months (95% CI 1.2-not estimated [NE]), NE (95% CI 5.6-NE), and 5.6 months (95% CI 2.6–8.3) for the 1, 3, and 5 mg/kg dose groups, respectively. The 6 and 12 month PFS rates were 57.0% (95% CI 33.2–75.1) and 28.5% (95% CI 10.7–49.5), respectively (Table 3). Regarding OS, the median OS for all participants was 15.9 months (95% CI 8.4-NE), as shown in Fig. 3B. This encompassed specific median OS durations of 11.1 months (95% CI 5.2-NE), NE (95% CI 7.2-NE), and NE (95% CI: 5.0-NE) for the 1, 3, and 5 mg/kg dose groups, respectively. The 6 and 12 month OS rates were 87.1% (95% CI 64.9–95.6) and 66.6% (95% CI 41.6–82.9) for all participants, respectively (Fig. 3B, Table 3).

### Biomarker analysis

Biomarker analysis samples were available from 11 participants, comprising five with SD and six with PR. The application of PCA and OPLS-DA revealed distinct metabolite clustering patterns between participants exhibiting PR and SD (Supplementary Figure S1A-B). This clustering was particularly evident in participants with PR. In terms of differentially expressed metabolites, there were 36 upregulated and 110 downregulated metabolites in participants with PR compared to those with SD (Supplementary Figure S2A). Notably, levels of SCFA including butyric, acetic, and valeric acid were significantly lower in participants with SD than in those with PR (all raw*P* < 0.01, Impact > 0, Supplementary Figure S2B). Metabolic pathway topology analysis highlighted the enrichment of propanoic and acetic acid. Furthermore, acetic acid emerged as a potential predictor of treatment response, displaying an AUC of 0.933 (95% CI 0.733–1) (Supplementary Figure S2C).

## Discussion

In the rapidly evolving landscape of immunotherapy, the emergence of dual-targeting immune checkpoint inhibitors has presented promising therapeutic avenues. These novel agents have consistently demonstrated a relatively benign safety profile, often comparable to their single-target antibody counterparts. Recent data from a phase I trial examining m7824, a bispecific agent targeting PD-L1 and TGFΒ in advanced solid tumors, revealed TRAEs of grade ≥ 3 in 21.1% of participants, encompassing conditions such as skin infections, asymptomatic lipase elevation, colitis coupled with anemia, and gastroparesis accompanied by hypokalemia [20]. Another trial involving Bintrafusp alfa, a PD-L1/TGFΒ fusion protein, in ESCC yielded TRAEs in 63.3% of participants, with 23.3% experiencing grade ≥ 3 events [21]. Our current investigation revealed an encouraging safety profile for KN046. During the dose-escalation phase, no DLT events were detected. Moreover, the incidence of grade ≥ 3 KN046-associated TEAEs was observed at 16.0%, lower than reports from other studies involving the combination of nivolumab and ipilimumab [3]. These results suggested the advantage of bispecific antibodies, like KN046, over combinations of two monoclonal antibodies. This incidence also aligns favorably with data from single-target anti-PD-L1 antibodies [22], indicating that AEs associated with KN046 remain manageable in the context of ESCC. Of particular note, the reported incidences of KN046-related SAEs and treatment discontinuations in our study, 24% and 16.0% respectively, mirror findings from prior studies on dual checkpoint inhibitors [20, 21]. It's also worth emphasizing that the integration of KN046 did not elicit unexpected safety concerns when juxtaposed against the safety profile of chemotherapy plus radiotherapy for advanced ESCC [23].

The synergy between radiotherapy and immunotherapy is of pivotal importance in contemporary oncologic research [6, 7]. Central to this hypothesis is the abscopal effect, wherein local radiotherapy induces systemic anti-cancer responses beyond the irradiated field [8, 9]. Radiotherapy likely boosts the systemic release of tumor-associated antigens, subsequently taken up by antigen-presenting cells and presented to CD8 cytotoxic T lymphocytes, culminating in a systemic anti-tumor response [10]. Moreover, radiotherapy may modulate the tumor microenvironment to be more receptive to T cell infiltration, reducing inhibitory signals like transforming growth factor β and thereby making the tumor more susceptible to immune cells [24, 25]. Clinically, this promise is being realized. In metastatic non-small-cell lung cancer, integrating pembrolizumab immunotherapy with radiotherapy significantly improved patient outcomes [12]. Likewise, the a phase 2 trial combining stereotactic body radiotherapy with pembrolizumab and trametinib emerged as a promising approach for recurrent post-surgery pancreatic cancer patients [13]. Our study sought to extend this investigation to the context of ESCC, combining chemotherapy, palliative radiotherapy, and subsequent administration of KN046.

At the outset of this study, first-line treatments for ESCC did not predominantly include immunotherapy-chemotherapy combinations or dual immunotherapy. Our exploration into combining KN046 with chemotherapy and palliative radiotherapy addressed a pressing need to identify more efficacious treatments for advanced ESCC. Since our study’s inception, both strategies have been approved as frontline treatments, underscoring the fast-evolving nature of oncological interventions. The pivotal trials such as KEYNOTE-590 [2] and CheckMate 648 [3] have underscored the benefits of adding agents like pembrolizumab or nivolumab to chemotherapy for patients with advanced ESCC. A median PFS of approximately 6 months and a median OS of around 13 months were observed. Another phase II trial juxtaposing camrelizumab with apatinib and chemotherapy revealed a median PFS of 6.85 months and a median OS of 19.43 months [26], thereby emphasizing the viability of immunotherapy combinations. Concurrently, the CheckMate 648 trial discerned the benefits of nivolumab and ipilimumab in prolonging the OS of patients with advanced ESCC, although no PFS benefits were noted in comparison to chemotherapy alone [3]. Our findings, revealing a median PFS and OS of 7.8 and 15.9 months respectively, suggest that KN046 holds comparable efficacy to other immunotherapy regimens, even those harnessing dual immunotherapeutic agents, in advanced ESCC. Moreover, our research postulates that palliative radiotherapy might further bolster the efficacy of immune checkpoint inhibitors, given its potential influence on the tumor microenvironment. This assertion was also evidenced by previous trials involving camrelizumab plus radiotherapy [27], camrelizumab plus chemoradiotherapy [28], atezolizumab plus chemoradiotherapy [29] and sintilimab plus chemoradiotherapy [30] in esophageal cancers. While the disparities due to study population heterogeneity might exist, the precise role of radiotherapy in augmenting the action of immune checkpoint inhibitors warrants further exploration in subsequent studies.

The relationship between the gut metabolome and the efficacy of immunotherapy remains a subject of ongoing discussion. Metabolites like SCFAs play a crucial role in mediating interactions among diet, microbiota, and the host. Emerging evidence has indicated links between SCFAs and treatment responses to pembrolizumab and nivolumab, as well as prognoses in patients with solid tumors [31]. SCFAs affect the CD4^+^ T cells, with butyric acid being able to induce FOXP3^+^ CD4^+^ Tregs differentiation [32–34]. SCFAs can also inhibit histone deacytelases [35–37], which inhibit CD4^+^ T cell apoptosis, upregulate antitumor immune response, and suppress tumor growth [38–40]. Gut-derived SCFAs influence the tumor response and the occurrence of AEs [41]. In this study, discernible differences in SCFA levels were observed between patients with SD and PR, particularly in the case of acetic acid, which could potentially serve as a predictive marker for treatment response. It's worth noting, however, that the sample size for gut metabolome and microbiota analysis was relatively modest. Given the variations in racial backgrounds, dietary habits, and cultural factors, further research into the relationship between the gut metabolome, microbiota, and treatment responses is warranted.

Our study, being a phase I trial, inherently bears certain limitations. Principally, the limited sample size restricts our capacity to draw statistically significant conclusions. The data on efficacy, while encouraging, remains preliminary, with the study's short-term follow-up serving as an additional constraint in its interpretability. The absence of comparator groups further inhibits our ability to make robust comparisons of the therapeutic efficacy and safety of KN046. In addition, the individualized radiotherapy protocol might produce certain bias when interpreting the abscopal effect of radiotherapy. Due to the exploratory nature and small sample size of this phase Ib trial, comprehensive biomarker analyses including tissue-derived or blood-derived markers such as PD-L1 were not conducted. It is imperative, hence, that subsequent investigations expand upon these findings through head-to-head studies, encompassing larger patient cohorts and extended follow-up periods to circumvent the aforementioned limitations.

In this phase I trial, the combination of KN046, a dual immune checkpoint inhibitor targeting PD-L1 and CTLA-4, with chemotherapy and palliative radiotherapy has shown feasibility for patients with advanced ESCC, exhibiting a commendable safety profile. Intriguingly, our findings also hint at the potential role of the gut’s metabolomic and microbiotic profile as predictive markers for treatment response.

## Supplementary Information

Below is the link to the electronic supplementary material.

Supplementary file1 (DOCX 467 KB)

## Data Availability

All data relevant to the study are included in the article or uploaded as supplementary information.
