# Supplementary material for: First-line treatment with KN046, chemotherapy and palliative radiotherapy for advanced esophageal squamous cell carcinoma: an open-label, dose escalation, and dose expansion phase Ib trial
Source: Cancer Immunol Immunother. 2024 Aug 6;73(10):194. doi: 10.1007/s00262-024-03769-4 (PMC11303366; doi:10.1007/s00262-024-03769-4)
Supplement: Supplementary file 1 — Supplementary file1 (DOCX 467 KB) [file 262_2024_3769_MOESM1_ESM.docx]

***Supplementary Material***

**First-line Treatment with KN046, Chemotherapy and Palliative Radiotherapy for Advanced Esophageal Squamous Cell Carcinoma: An Open-label, Dose Escalation, and Dose Expansion Phase Ib Trial**

**Content**

[Supplementary Table S1. Inclusion and exclusion criteria 2](#_Toc145504271)

[Supplementary Table S2. Criteria for dose-limiting toxicity 7](#_Toc145504272)

[Supplementary Table S3. Adverse events during dose-escalation period 9](#_Toc145504273)

[Supplementary Figure S1. Principal component analysis (PCA), orthogonal projections to latent structures discriminant analysis (OPLS-DA) of metabolite. 10](#_Toc145504274)

[Supplementary Figure S2. Metabolite profile (A), enriched metabolite (B) of patients with stable disease (n=5) and partial response (n=6), and the predictor of treatment response (C). 11](#_Toc145504275)

# Supplementary Table S1. Inclusion and exclusion criteria

| Enrollment criteria:   1. Age ≥18 and ≤75 years old, no gender preference; 2. Understood the informed consent and were willing to participate in this study and signed the informed consent; 3. Histologically-confirmed recurrent/metastatic ESCC: 4. No systematic treatment for recurrent/metastatic ESCC was received; 5. Previous experience with neoadjuvant or adjuvant chemotherapy (including chemotherapy or chemoradiotherapy) was allowed, but such treatment should be terminated 6 months (180 days) prior to this study; 6. There were indications of radiotherapy of the primary lesion and local lymph node lesions or metastatic lesions of ECC, such as the alleviation of dysphagia, pain, and/or gastrointestinal bleeding. 7. In accordance with the RECIST 1.1 guideline, there was at least one measurable lesion at baseline. If there was only one measurable lesion at baseline, this lesion must not have received prior radiotherapy, or evidence suggested obvious progression in this lesion at the end of radiotherapy; 8. Formalin-fixed paraffin-embedded or unstained tumor tissue slides (prepared within 1 week) were recently obtained (biopsy specimens from a non-radioactive region within two years), suitable for the tissue determination of biomarkers, and containing tumor tissues; 9. The PS score of 0-1; 10. Liver function met the following criteria within seven days before the first administration: 11. Total bilirubin ≤1.5 × ULN (in case of Gilbert syndrome, total bilirubin ≤ 3 × upper limits of normal [ULN]); 12. Transaminases (ALT/AST) ≤2.5 × ULN (in case of a subject with liver metastases ≤3 × ULN); 13. Renal function met the following criteria within seven days before the first administration: Serum creatinine ≤1.5 × ULN and the serum creatinine clearance rate ≥60 mL/min (calculated with the Cockcroft-Gault formula); 14. Bone marrow function met the following criteria within seven days before the first administration (subjects were not allowed to receive a blood transfusion or growth factors because neutrophil count, blood platelet count (BPC), or hemoglobin was lower than the relevant research requirements within seven days before the laboratory examination): 15. Hemoglobin ≥9.0 g/dL; 16. Absolute neutrophil count (ANC) ≥1.5 × 10^9^/L; 17. Platelet ≥100 × 10^9^/L; 18. INR or PT ≤1.5 × ULN, and aPTT or PTT ≤1.5 × ULN. 19. Life expectancy ≥3 months; 20. Fertile female subjects or male subjects with a fertile partner agreed to adopt highly effective contraceptive measures seven days before the first administration till 24 weeks after the first administration. The results of the blood hCG pregnancy test of fertile female subjects within seven days before the first administration must be negative; 21. Able and willing to conform to the procedures specified in the study protocol, such as follow-up visits, the treatment plan, and lab examinations. |
| --- |
| Exclusion criteria:   1. Untreated active brain metastases or meningeal metastases. If the metastatic brain lesion of a subject had been treated, and the metastatic lesion was stable (the brain imaging examination before the first administration of KN046 showed that the lesion had been stable for at least four weeks without new neurological symptoms, or neurological symptoms had recovered to the baseline) without evidence of a new brain metastatic lesion or increased in the original brain metastatic lesion, the subject could be enrolled; 2. Participated in any other clinical trials within 28 days before the first administration; 3. Another anti-tumor treatment not specified in the study protocol was received within 28 days before the first administration; 4. Major surgical treatment (e.g., transabdominal or transthoracic surgery, excluding diagnostic puncture or venous catheterization) was received within 28 days before the first administration; 5. Radical radiotherapy was received three months before the first administration. Palliative radiotherapy was received two weeks before enrollment. 6. Prior treatment with immune checkpoint blockers or T-cell costimulation blockers, including but not limited to immune checkpoint blockers and therapeutic vaccines, such as PD-1, PD-L1, CTLA-4, and LAG-3; 7. Systematic glucocorticoids (> 10 mg/day prednisone or other glucocorticoids in the equivalent amount) or immunosuppressants were needed within 14 days before the first administration. Inhalation or local application of hormones, preventive medication, and hormonotherapy in an alternative biological dose due to adrenal insufficiency were excluded. Short-term (≤ seven days) corticosteroids for the prevention (e.g., contrast reactions) or treatment of non-autoimmunity diseases (e.g., delayed-type hypersensitivity due to contact of allergens) were accepted; 8. Live vaccines (including live-attenuated vaccines) were vaccinated within 28 days before the first administration; 9. Interstitial pneumonia/interstitial lung disease in the past or at the time of enrollment; 10. Autoimmune diseases in the past or at the time of enrollment, including but not limited to Crohn's disease, ulcerative colitis, systemic lupus erythematosus, nodule diseases, Wegener's syndrome (granulomatosis with polyangiitis, Graves' disease, rheumatoid arthritis, hypophysitis, and uveitis), autoimmune hepatitis, systemic sclerosis (e.g., scleroderma), Hashimoto's thyroiditis (see exceptions below), autoimmune vasculitis, and autonomic neuropathy). However, the following circumstances were excluded: Type 1 diabetes, stable hypothyroidism under hormone replacement therapy (including hypothyroidism caused by autoimmune thyroid disease), psoriasis not requiring systemic therapy, or leukoderma; 11. Complications of other malignant tumors within 5 years before the first administration. Cured cutaneous squamous cell carcinoma, basal cell carcinoma, non-invasive superficial bladder cancer, low-risk localized prostatic cancer in situ (defined as: Grade ≤T2a, the Gleason score ≤6 points, and PSA ≤10 ng/mL (if measured) suggested in the diagnosis of prostate cancer. Subjects who received radical treatment without prostate-specific antigen (PSA) biochemical relapse could participate in this study), and prostatic cancer/cervical cancer/breast cancer in situ was excluded; 12. Uncontrolled complications, including but not limited to the following circumstances: 13. Active phase of HBV or HCV infection. HBsAg-positive subjects should take a test of HBV DNA. If HBV DNA >500 IU/mL (or 2500 copies/mL), the subjects should be excluded. HBsAg-positive patients could be enrolled if HBV-DNA ≤500 IU/mL (or 2500 copies/mL) after antiviral treatment. HCV antibody-positive subjects were excluded; 14. Known HIV infection or medical history of AIDS; 15. Active tuberculosis; 16. Patients had an active infection or systematic application of anti-infective drugs for more than one consecutive week within 28 days before the first administration in this study; 17. Patients had uncontrollable hypertension (resting blood pressure ≥ 150/95 mmHg), symptomatic cardiac insufficiency (NYHA II-IV), unstable angina pectoris, or myocardial infarction within six months, or a prolonged QTc interval or a risk of arrhythmia (baseline: QTc >470 ms < Fridericia QT correction>, irreformable hypokalemia, long QT syndrome (LQTS), atrial fibrillation or severe valvular disease with the resting heart rate >100 bpm, and history of arrhythmia of significance); 18. Active bleeding. 19. The toxicity of prior anti-tumor treatment did not recover to CTCAE grade ≤1 (NCI-CTCAE v5.0) or the baseline, excluding baldness (any grade was accepted); 20. History of allogeneic bone marrow transplantation or organ transplantation; 21. History of allergies, hypersensitive reactions, or intolerance to antibody drugs; history of obvious drug allergies (e.g., serious allergic reactions, immune-mediated hepatotoxicity, immune-mediated thrombocytopenia (IMTP), or anemia); 22. Women who were pregnant and/or in the lactation period; 23. History of fistulas (esophagus/bronchi or esophagus/aortas) or possible fistulas; 24. Within 1 month prior to randomness, unintentional weight loss ≥ 5% or other indicators suggested severe malnutrition; 25. Other circumstances that the researchers believed would influence the safety or compliance of drugs in this study, including but not limited to a medium to a large amount of pleural effusion/ascites/hydropericardium, irreformable pleural effusion/ascites/hydropericardium (relapse within two weeks after interventions), and mental disorders. |

# Supplementary Table S2. Criteria for dose-limiting toxicity

| Hematologic toxicity:   1. grade ≥3 neutropenia lasting >7 days 2. febrile neutropenia 3. grade ≥3 thrombocytopenia with a bleeding tendency or requiring platelet transfusion 4. grade ≥4 hematologic toxicity, except for solitary grade 4 lymphocytopenia. |
| --- |
| Non-hematologic toxicity:   1. grade ≥3 elevated serum creatinine 2. grade ≥3 total bilirubin elevation 3. grade 3 alanine aminotransferase (ALT) and/or aspartate aminotransferase (AST) elevation that persisted for >7 days 4. grade 4 ALT and/or AST elevation, 5. grade ≥3 central nervous system (CNS) toxicity 6. grade ≥3 cardiotoxicity, 7. grade ≥3 asymptomatic serum pancreatic amylase or lipase elevation that persisted for >14 days 8. serum pancreatic amylase or lipase elevation accompanied by clinical signs and symptoms requiring medical intervention. 9. any grade ≥3 non-hematologic event, except: 10. alopecia of any grade 11. transient (≤72 h) grade 3 fatigue, local reactions, headache, nausea, and vomiting that resolved to grade ≤1 12. grade 3 diarrhea, grade 3 oral/gastric mucositis, grade 3 skin toxicity, grade 3 elevated liver function (ALT, AST, or γ-glutaryl transferase (GGT)) that resolved to grade ≤1 within 7 days of medical intervention 13. grade 3 infusion-related reactions that resolved within 6 h of medical intervention 14. grade 3 influenza-like symptoms or brief fever (≤6 h) after medical intervention 15. single laboratory test abnormalities that were not clinically relevant and resolved to grade ≤1 within 7 days after appropriate medical intervention 16. tumor relapse phenomenon manifesting as pain, local irritation, or local rash localized due to a known or suspected tumor. |

# Supplementary Table S3. Adverse events during dose-escalation period

|  | All (n=9) | 1 mg/kg (n=3) | 3 mg/kg (n=3) | 5 mg/kg (n=3) |
| --- | --- | --- | --- | --- |
| Any TEAE | 9 (100.0) | 3 (100.0) | 3 (100.0) | 3 (100.0) |
| KN046-related TEAE | 4 (44.4) | 1 (33.3) | 2 (66.7) | 1 (33.3) |
| Grade ≥3 TEAE | 8 (88.9) | 3 (100.0) | 3 (100.0) | 2 (66.7) |
| Grade ≥3 KN046-related TEAE | 2 (22.2) | 1 (33.3) | 1 (33.3) | 0 |
| SAE | 6 (66.7) | 3 (100.0) | 3 (100.0) | 0 |
| KN046-related SAE | 2 (22.2) | 1 (33.3) | 1 (33.3) | 0 |
| KN046-related treatment discontinuation | 2 (22.2) | 1 (33.3) | 1 (33.3) | 0 |
| Most common TEAE | | | | |
| Leukopenia | 8 (88.9) | 3 (100.0) | 3 (100.0) | 2 (66.7) |
| Neutropenia | 7 (77.8) | 3 (100.0) | 3 (100.0) | 1 (33.3) |
| Thrombocytopenia | 6 (66.7) | 2 (66.7) | 2 (66.7) | 2 (66.7) |
| Nausea | 5 (55.6) | 3 (100.0) | 2 (66.7) | 0 |
| Anemia | 4 (44.4) | 3 (100.0) | 1 (33.3) | 0 |
| Most common SAE |  |  |  |  |
| Leukopenia | 7 (77.8) | 2 (66.7) | 3 (100.0) | 2 (66.7) |
| Neutropenia | 7 (77.8) | 3 (100.0) | 3 (100.0) | 1 (33.3) |

TEAE: treatment-emergent adverse events; SAE: serious adverse event.

**
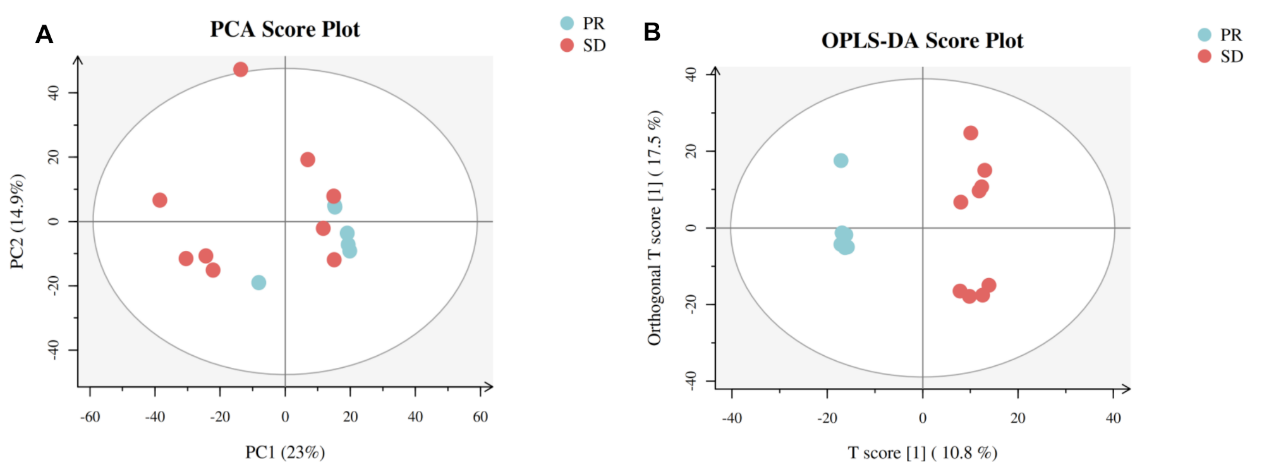
**

# Supplementary Figure S1. Principal component analysis (PCA), orthogonal projections to latent structures discriminant analysis (OPLS-DA) of metabolite.


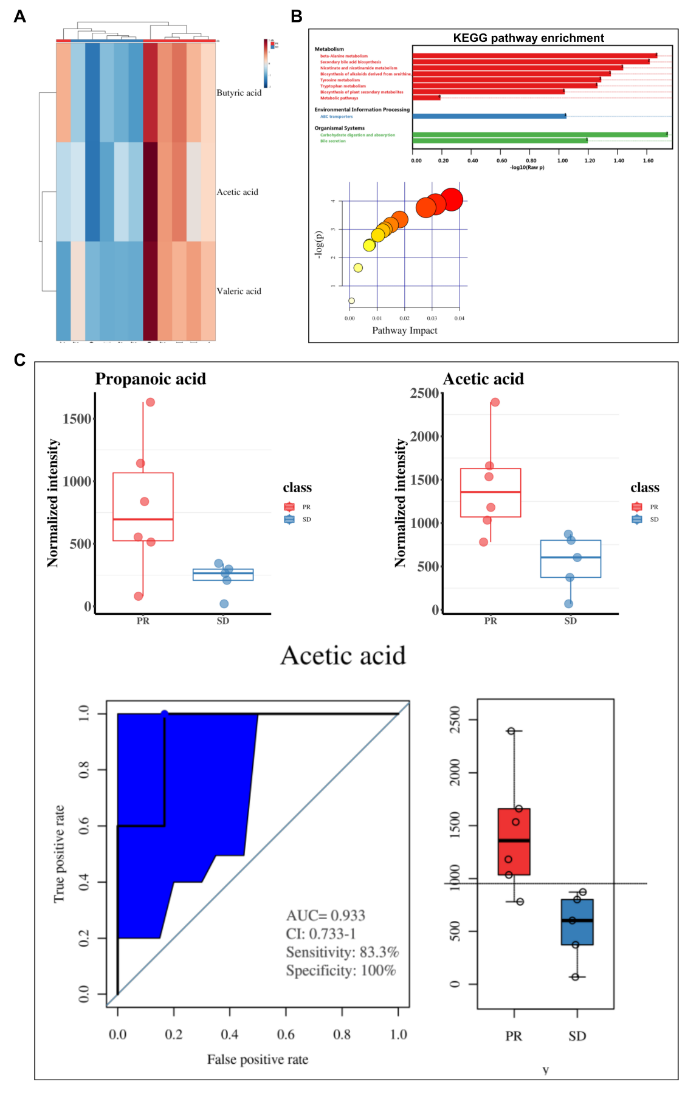


# Supplementary Figure S2. Metabolite profile (A), enriched metabolite (B) of patients with stable disease (n=5) and partial response (n=6), and the predictor of treatment response (C).
